# Effects of Hygrothermal and Salt Mist Ageing on the Properties of Epoxy Resins and Their Composites

**DOI:** 10.3390/polym15030725

**Published:** 2023-01-31

**Authors:** Baoming Wang, Shengzong Ci, Mingzhe Zhou, Chengrui Di, Junwei Yu, Bo Zhu, Kun Qiao

**Affiliations:** 1 School of Materials Science and Engineering, Shandong University, Jinan 250012, China; 2 School of Electromechanical and Information Engineering, Shandong University, Weihai 264209, China; 3 School of Metallurgy and Materials, University of Birmingham, Edgbaston, Birmingham B15 2TT, UK

**Keywords:** polymer-matrix composites (PMCs), environmental degradation, moisture, pultrusion, moisture absorption

## Abstract

Epoxy and epoxide composites have a wide range of outdoor applications wherein they are affected by ageing. In this study, epoxy casting plates and epoxy-based composite rods for use in overhead conductors were prepared. A concurrent investigation concerning the ageing of epoxy resins and their carbon fibre composites was carried out via artificially accelerated experiments under hygrothermal and salt mist conditions. The moisture penetration along the depth, water absorption, appearance, hardness, density of the epoxy resins, and variation patterns of the impact strength and tensile strength of the epoxy-based composites were investigated. The ageing mechanisms were explored using Fourier transform infrared spectroscopy (FTIR) and scanning electron microscopy (SEM). Both ageing modes had essentially similar influences on the properties of the resins and their composites; moreover, they did not significantly affect the chemical structure and microstructure of the epoxy resin, with the physical adsorption of water primarily observed during the ageing process. The moisture absorption behaviour of the epoxy obeyed Fick’s law. Although the water penetration rate in the salt mist ageing mode was slightly higher than that in the hygrothermal ageing mode during the early ageing stage, it was essentially the same during the later stage. The final moisture absorption rate at saturation was approximately 1.1% under both modes. The flexural strengths and impact strengths of the composites in both ageing modes followed a similar trend. They decreased gradually with the ageing time and then stabilized at almost the same value. The flexural strength was reduced from 803 MPa to 760 MPa and the impact strength from 383 J/m^2^ to 310 J/m^2^, indicating a decrease of approximately 5.4% and 19%, respectively. The absorbed water during the ageing process caused micro-cracks at the interface between the fibres and resin, weakening the interfacial strength and reducing the mechanical properties of the composites.

## 1. Introduction

Epoxy resins and their composite materials exhibit remarkable mechanical properties, electrical insulation properties, and chemical resistance, and they have been widely used in many industries, such as the automobile, electronics, packaging, sports, and 3D printing industries. Moreover, in some engineering applications, they have gradually replaced some traditional metal materials. Typically, the composite parts of some large-scale applications, such as aircrafts, watercrafts, wind turbine blades, and offshore structures, are huge [1,2,3,4,5].

However, there are some inconvenient issues associated with composites regarding their long-term performance and environmental durability. In some applications of composites, especially outdoor applications, due to the influence of high temperature, humidity, salt mist, ultraviolet, and other environmental factors [6,7,8,9], ageing will inevitably occur, leading to material performance degradation and even failure, thereby reducing the reliability and safety of products. Therefore, a comprehensive understanding of the ageing mechanisms and environmental deterioration of composites is of great significance in relation to predicting the service life and durability of composites.

Over the past few decades, a series of studies have been conducted to prove that ageing can affect both the physical and mechanical properties of composite materials [10,11,12,13,14,15]. Reportedly, the mechanical properties of composites are reduced in hygrothermal and salt mist environments, primarily due to moisture absorption [6,16,17,18]. It is well known that water absorption may cause numerous unwanted effects, such as swelling, plasticization, and degradation of mechanical, electrical, or other properties [19,20,21,22,23,24,25,26,27,28]. For example, Alessandro et al. [29] found that the tensile strength and apparent fracture toughness of a recycled poly(ethylene terephthalate) matrix and its composites decreased noticeably under hygrothermal conditions. Liu et al. [30] studied the fundamental structure–property relationships of poly[2,2′-(m-phenylene)-5,5′-bibenzimidazole] (PBI) affected by higher temperature and high moisture content environments, and they revealed that the brittle fracture nature of PBI is caused by the hot water-induced degradation of the PBI’s molecules. Numerous studies have been carried out to explain these phenomena [31,32]. However, there is no universal model to support the notion that all water absorption and diffusion phenomena are due to the complexity of the molecular interactions between the matrix and water [33]. Some researchers have proposed the existence of two types of absorbed water molecules: free and bound water [34,35]. Other researchers have suggested that there are three different modes of water absorption in a resin system [36,37]. Currently, it is recognized that the best approaches for studying the water absorption of resins are based on Fick’s law [38,39,40,41] and the Langmuir model [42,43].

In addition, the effect is not only on the resin matrix, but also on the interphase between the fibres and resins [44,45]. The performance of composites is largely dependent on the fibre/matrix interface. Hygrothermal exposure would degrade the interfacial bonding of the composites and lead to the deterioration of the properties of the composites. In turn, the weakening of the fibre/matrix interface would promote the diffusion of water into the debonded area [46]. Jarrett et al. [47] studied the interlaminar shear strength and glass transition temperature of a carbon fibre/epoxy composite after it was immersed in water at different temperatures until partially saturated.

The ageing properties of epoxy resin composites primarily result from the ageing of the resin matrix and the ageing of the composite interface; therefore, the ageing properties of epoxy resins and their composites need to be studied simultaneously.

**Figure 1:**
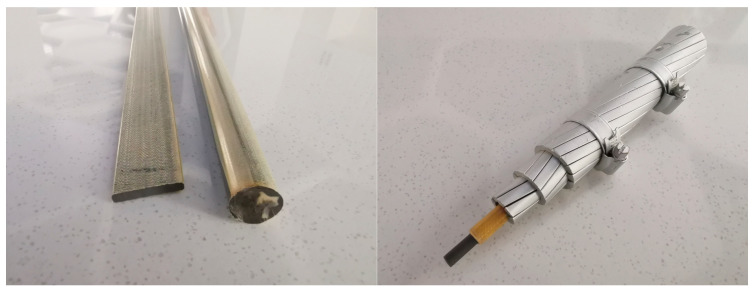
Carbon fibre sucker rod and conductor core.

At present, few studies have presented a concurrent investigation of the ageing of epoxy resins and epoxy resin-based composites. The research on the ageing performance of epoxy resins is mainly focused on coatings and other thin-layer products (thickness less than 1 mm), thereby lacking insight into the performance of comparatively thick epoxy resins across the thickness [2], especially in the case of an epoxy/anhydride system. With the technological development of composite materials, epoxy resins and their composites have been transitioning into larger and thicker products. An epoxy resin that has been successfully used in carbon fibre conductor cores and sucker rods has been reported in previous studies [48,49]. Unlike coated products, their thickness can be up to 5–10 mm or more, as shown in Figure 1. The application time of carbon fibre sucker rods and carbon fibre conductors is still short, meaning that the research on their properties, especially the research on the properties of their long-term operation under actual complex conditions, is basically at the initial stage or even non-existent, which results in a lack of guiding data on the performance grasp and operation maintenance of products in actual operation. Their long-term performance in complex and harsh natural environments, such as high-temperature, hygrothermal, and salt mist environments, is still unknown. Hence, the study of the ageing performance and ageing patterns of epoxy resins and relevant composite materials with certain thicknesses is critical to ensuring the safety and promotion of composite products.

This study compared the moisture absorption behaviour of epoxy resins across the thickness as well as the changes in the physical properties, thermal properties, chemical structure, and microstructure under artificially accelerated hygrothermal and salt mist ageing conditions. We investigated the evolution of the mechanical properties and microstructure of carbon fibre/glass fibre hybrid reinforced epoxy resin pultruded composites to explore the ageing pattern of epoxy resins and their composites, thereby laying the foundation for the manufacturing and safe application of composite materials. It should be noted that, in addition to the humidity and salt studied in this research, other factors, such as the temperature [50], pH [23], stress state [51], void contents [52], fibre orientation [53], and volume fraction [54], have a coupling effect on the ageing properties of epoxy and its composites.

## 2. Materials and Methods

### 2.1. Materials

An epoxy resin with multi-functional groups and an anhydride curing agent were purchased from Dow Chemical Company, Midland, MI, USA. The six-membered heterocyclic benzo compounds, toughening modifier for the “sea-island” structure, defoamer, release agent, and other reagents were developed at the Carbon Fiber Engineering Technology Research Centre of Shandong University, China. The carbon fibre (T700-12K-50C) was procured from Toray Industries, Inc., Tokyo, Japan. The high-strength bidirectional glass fibre tape was purchased from Shandong Lufa Carbon Fiber Composites Co., Jinan, China. The other reagents, namely sodium chloride, anhydrous ethanol, and acetone, were commercially purchased in China.

### 2.2. Sample Preparation

#### 2.2.1. Preparation of Epoxy Resin Castings

The epoxy resin, curing agent, modifier, and defoamer were mixed in fixed weight proportions of 100:100:30:6, respectively, to form a gel with a viscosity of approximately 300~500 mPa·s (25 °C). The gel was then poured into a mould and moulded into a 3 mm thick board through a series of processes, including vacuum defoaming, curing, cooling, and demoulding. The resulting epoxy resin board was cut into sample strips. The epoxy resins used in the hygrothermal and salt mist ageing study were cut from the same casting board to ensure uniformity in their performance and colour. The resin curing conditions were 80 °C/1 h + 100 °C/2 h + 160 °C/2 h.

#### 2.2.2. Preparation of Carbon Fibre/Glass Fibre Hybrid Reinforced Epoxy Pultruded Composites

**Figure 2:**
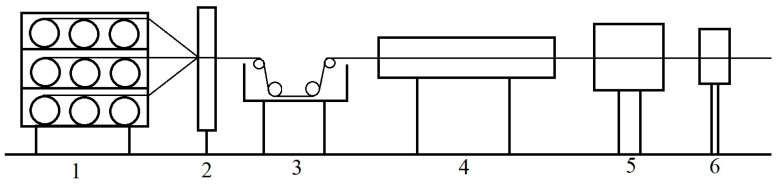
Schematic of pultrusion moulding (1. creel; 2. fibre guide; 3. resin impregnator; 4. mould; 5. tractor; and 6. cutter).

Composite rods with a diameter of 7.5 mm were prepared using a pultruding process. The epoxy resin used to prepare the composites was the same as that used in the preparation of the epoxy castings. The reinforcing fibres included internal unidirectional T700 carbon fibre and external high-strength bidirectional glass fibre cloth, and their volume content was 58% and 2%, respectively. The process, as schematically shown in Figure 2, was as follows: the carbon fibres and glass fibre tape were impregnated in an epoxy resin gel solution under the force of a pulling machine and then cured in a mould.

The curing temperature was 150~200 °C, and the pultrusion speed was 500 mm/min. The epoxy and its composite have previously been used in heat-resistant aluminium alloy composite core conductors [55].

### 2.3. Experimental Methods

#### 2.3.1. Hygrothermal Ageing

The hygrothermal ageing experiment was carried out according to the Chinese standard test, “Determination of the effects of humidity and heat, water spray, and salt mist on plastics” (GB/T 12000-2003), with alternating high- and low-temperature hygrothermal ageing conditions of (25 ± 3) °C × 12 h (humidity not less than 95%) and (55 ± 1) °C × 12 h (humidity maintained at (93 ± 3)%), respectively.

#### 2.3.2. Salt Mist Ageing

The salt mist ageing test was also carried out according to the Chinese standard test, “Determination of the effects of humidity and heat, water spray, and salt mist on plastics” (GB/T 12000-2003), with the salt mist ageing conditions listed in Table 1.

#### 2.3.3. Ageing Time

The epoxy resins and their composites were subjected to salt mist ageing and hygrothermal ageing for the same duration of time. The cycling period was 24 h. The ageing test evaluation time points are shown in Table 2.

### 2.4. Characterisation Methods

An electronic balance (AG204, Mettler Toledo) with a readability of 0.1 mg was used to measure the rate of the change in the mass of the epoxy resin castings. The resin density was measured using the buoyancy method. A digital microscope was used to observe the depth of the water penetration and the change in the colour of the epoxy resin castings. A Richter hardness tester (AR936, Sigma Technology) was used to determine the hardness of the epoxy resin. The structural changes in the epoxy resin castings were examined via Fourier transform infrared spectroscopy (VERTEX-70, Bruker) at a resolution of 4 cm^−1^ and 32 scans from 4000~370 cm^−1^. The FTIR samples were prepared via the potassium bromide method. The resin powder was ground from the surface located at almost the mid-plane of the aged sample and then mixed with KBr at a mass ratio of 1:100 to form small pieces of pellets. The thermal decomposition of the epoxy resin was analysed using a thermogravimetric analyser (SDT-Q600, TA Instruments) under the following conditions: heated under a nitrogen purge from 25 °C to 800 °C at a rate of 10 °C/min. The microstructural and morphological characterization of the epoxy resins and their composites was carried out using a scanning electron microscope (SEM, SU-70, Hitachi). A universal testing machine (CMT4204, Shenzhen Sans Testing Machine Co., Ltd., Shenzhen, China) was used to perform the three-point bending test on the composites. The loading speed was 2 mm/min, the length of the test samples was 120 mm, and the support span was 60 mm. An impact tester (XJJ-50) was used to perform the unnotched impact test on the epoxy resin-based composites with a pendulum energy of 15 J and a support span of 55 mm. The impact test here was for the composite conductor core product. The impact strength can be expressed by the following formula:

$$\mathrm{Impact}\;\mathrm{strength}=\frac{E}{\pi \times r^{2}}\times 10^{3}$$

where *E* is the impact energy absorbed by the specimen/J and *r* is the radius of the sample.

The relevant mechanical property tests in this paper were usually measured 5 times, and the average value of these measurements was taken as the final result.

## 3. Results and Discussion

### 3.1. Ageing Properties of Epoxy Resin Castings

#### 3.1.1. Moisture Absorption

**Figure 3:**
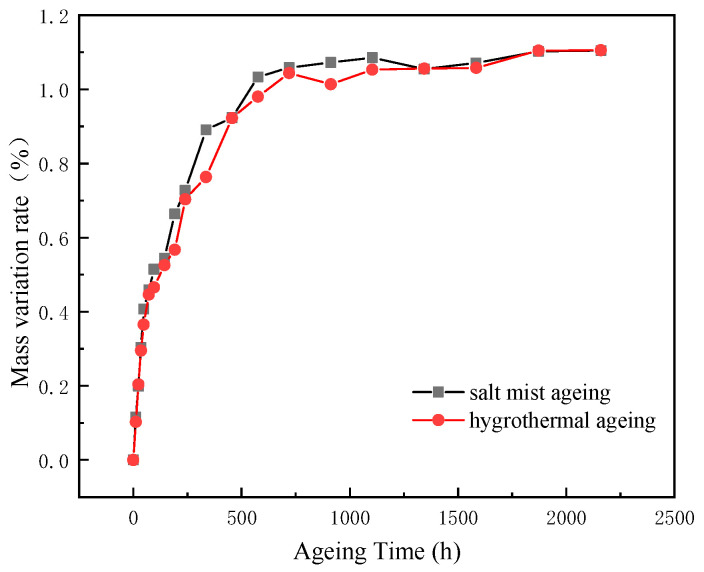
Rate of change in the mass of the epoxy resin during ageing.

Figure 3 shows the mass variation rates under the hygrothermal and salt mist ageing conditions. Both curves follow a similar trend. During the early stage of ageing, the mass variation rate (*Mt*) was almost a linear function of the ageing time, and the moisture absorption rate was relatively high. However, with an increase in the ageing time, the moisture absorption rate gradually decreased and eventually saturated. The moisture absorption rate in the salt mist ageing condition was slightly higher than that in the hygrothermal ageing condition. However, the final moisture absorption rate at saturation was approximately 1.1% under both conditions.

For the calculation of the moisture diffusion coefficient, Fickian theory is commonly employed, assuming uniform moisture and temperature conditions:

(1) $$\frac{\partial z}{\partial t}=D\frac{\partial^{2}C}{\partial z^{2}}$$

where *z* is the distance into the thickness (h) from an exposed surface, *C* is the concentration of water and *D* is the bulk diffusion coefficient (mm^2^/s).

The fractional moisture absorption of a plane sheet material can be expressed by the following formula:

(2) $$\frac{M_{t}}{M_{\infty }}=1-\frac{8}{\pi^{2}}\overset{\infty }{\underset{n=0}{\sum }}\frac{1}{{\left(2n+1\right)}^{2}}exp\left\{-{\left(2n+1\right)}^{2}\pi^{2}\left(\frac{Dt}{h^{2}}\right)\right\}$$

where *t* is the time the samples were immersed in water, $M_{t}$ is the quantity of water absorption during time *t* and $M_{\infty }$ is the corresponding quantity of water absorption during the infinite time, i.e., the saturation level. Moreover, *D* is the diffusion coefficient and *h* is the whole thickness of the plane sheet (3 mm).

As expressed by the above equations, the moisture absorption in single-phase diffusion can be expressed in terms of two parameters: *D* and $M_{\infty }$. They depend strongly on the nature of the material, relative humidity, and temperature.

Formula (2) can be simplified for short- and long-term time approximations as:

(3) $$\frac{M_{t}}{M_{\infty }}=\frac{4}{\pi^{2}}\sqrt{\frac{Dt}{h^{2}}}\;\mathrm{for}\;\frac{Dt}{h^{2}}<0.04$$

and

(4) $$\frac{M_{t}}{M_{\infty }}=1-\frac{8}{\pi^{2}}\exp\left(\frac{-Dt}{h^{2}}\pi^{2}\right)\;\mathrm{for}\;\frac{Dt}{h^{2}}>0.04$$

The diffusion coefficient *D* in the direction normal to the material surface can be computed by applying Formula (5) from the initial linear portion of the absorption curve using the long-term approximation according to Fick’s diffusion model:

(5) $$D=\pi {\left(\frac{h}{4M_{\infty }}\right)}^{2}S^{2}=\pi {\left(\frac{h}{4M_{\infty }}\right)}^{2}{\left(\frac{M_{2}-M_{1}}{\sqrt{t_{2}}-\sqrt{t_{1}}}\right)}^{2}$$

where *S* is the slope of the initial zone of the moisture absorption curve and *M*_1_ and *M*_2_ are the values of the moisture content at the arbitrary time *t*_1_ and *t*_2_ during the initial stage, respectively. The diffusion coefficients under the two ageing conditions are shown in Table 3.

**Figure 4:**
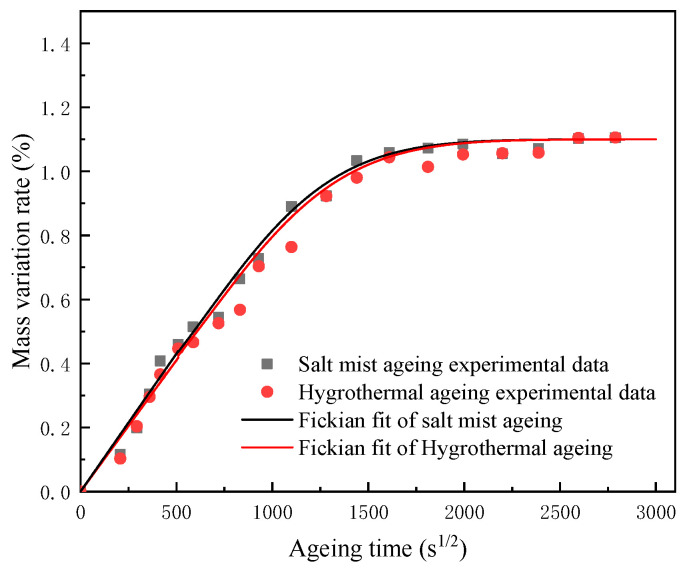
Fickian fit.

After plotting the results as shown in Figure 4, it was concluded that the moisture absorption behaviour of the epoxy obeyed Fick’s law.

The moisture absorption process of the epoxy resin mainly involved two aspects: the diffusion of water molecules into the resin matrix and the aggregation of water molecules in the pores, micro-cracks, and interfacial debonding defects. The water molecules entered the free voids in the resin via free diffusion. The absorbed water increased the distance between the matrix’s macromolecular structure, increased the activity of the rigid groups, and then produced plasticization. The absorbed water produced osmotic pressure that caused cracks, tiny cracks, or other types of morphological changes inside the epoxy resin to form water channels. The moisture absorption rate of the salt mist ageing condition was slightly higher than that of the wet heat ageing condition. The Cl^-^ and Na^+^ ions enhanced the diffusion ability of the water molecules in the epoxy resin.

**Figure 5:**
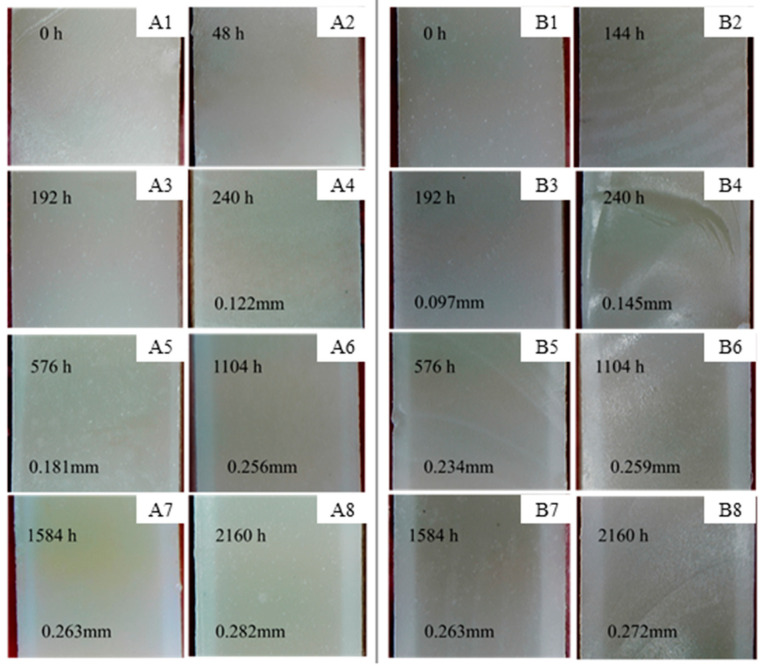
Cross-sectional images of the epoxy resin casting sheets after different ageing time ((**A1**–**A8**) hygrothermal ageing, (**A1**) 0 h, (**A2**) 48 h, (**A3**) 192 h, (**A4**) 240 h, (**A5**) 576 h, (**A6**) 1104 h, (**A7**) 1584 h, and (**A8**) 2160 h; (**B1**–**B8**) salt mist ageing, (**B1**) 0 h, (**B2**) 48 h, (**B3**) 192 h, (**B4**) 240 h, (**B5**) 576 h, (**B6**) 1104 h, (**B7**) 1584 h, and (**B8**) 2160 h).

Figure 5 shows cross-sectional images of the epoxy resins at different ageing times. The changes in the colour of the resins’ edges are evident. As the ageing time increased, distinct light-coloured “bands” appeared around the edges of the epoxy cross-sections, and the width of the bands gradually increased. The width of the band indicates the depth of the water penetration. As can be seen from Figure 5, the bands appeared earlier in the salt mist ageing condition (after 192 h), whereas they appeared after 240 h in the hygrothermal ageing condition. The bands’ width in the salt mist ageing condition was larger than that in the hygrothermal ageing condition at the same ageing time. This indicated that the rate of the moisture absorption by the epoxy resin in the salt mist ageing condition was slightly higher than that in the hygrothermal ageing condition under the test conditions. After 2160 h, the width of the bands under the two ageing modes was essentially similar, 0.282 mm (hygrothermal) and 0.272 mm (salt mist), indicating that the saturation moisture absorption rates of the epoxy resin in both modes were essentially the same. This is consistent with the results of the moisture absorption rate test shown in Figure 3.

**Figure 6:**
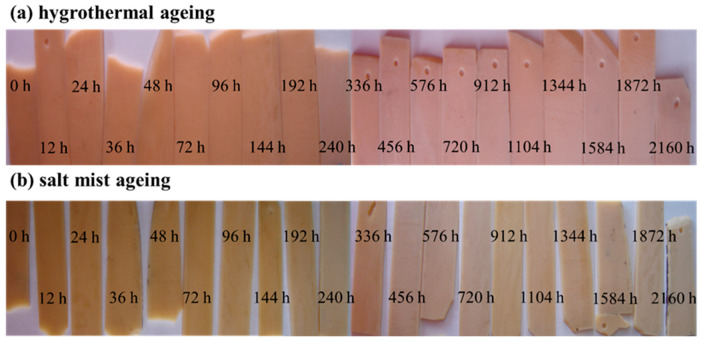
Change in the colour of the epoxy resin casting sheets before and after ageing: (**a**) hygrothermal ageing; and (**b**) salt mist ageing.

Figure 6 shows the changes in the colour of the epoxy resin before and after ageing. As the ageing time increased, the colour of the epoxy resin gradually faded, changing from yellowish brown to light white. In order to determine whether changes happened in the epoxy’s chemical structure during the ageing process, a functional group analysis of the resin on the ageing samples’ surface was performed via FTIR, and the results showed that there was no change, see Section 3.1.4 for detailsThe whitening phenomenon could be attributed to the scattering of light by the water molecules absorbed in the resin. The primary colour of epoxy resin is yellow. Since water molecules are small, long-wavelength electromagnetic waves, such as red light, can pass through water molecules, while short-wavelength electromagnetic waves, such as blue light, cannot pass through water molecules and so will be scattered. According to the complementary colour theory proposed by Ewald Herring, blue and yellow complement each other. When two complementary colours are mixed in appropriate proportions, a white colour will be produced. As a result, the ageing resin turned white, and as the ageing time increased, the water content in the resin gradually increased and the more blue light was scattered, meaning that the whitening became more significant. The colour change in the salt mist ageing samples occurred earlier (after 144 h) than that in the hygrothermal ageing samples (after 192 h). This confirms that the moisture absorption rate in the salt mist ageing samples was slightly higher than that in the hygrothermal ageing samples.

#### 3.1.2. Density

**Figure 7:**
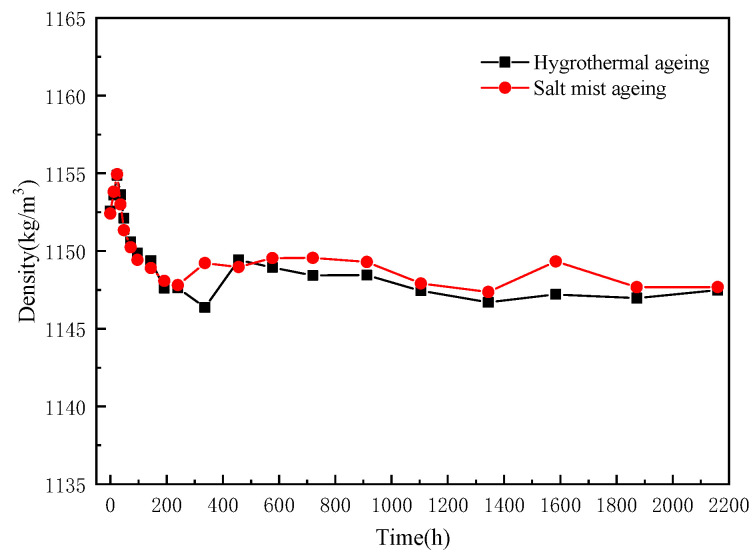
Changes in the density of the epoxy resins before and after ageing.

The changes in the density of the epoxy resins at different ageing times are shown in Figure 7. Initially, the density of the epoxy resin increased marginally, and then it started to decrease after approximately 36 h.

At the beginning of the ageing, the high water absorption led to a rapid increase in the mass, although the change in the volume of the resin lagged behind the change in the mass; therefore, the water absorption during the initial 36 h did not result in a considerable volume change. Thereafter, the resin began to expand in volume with the gradual absorption and accumulation of water, thereby exhibiting an initial increase and subsequent decrease in density. After 456 h, the density of the epoxy resins essentially stabilized, approaching a saturation state of water absorption, and no further increase in the volume was observed.

#### 3.1.3. Hardness

**Figure 8:**
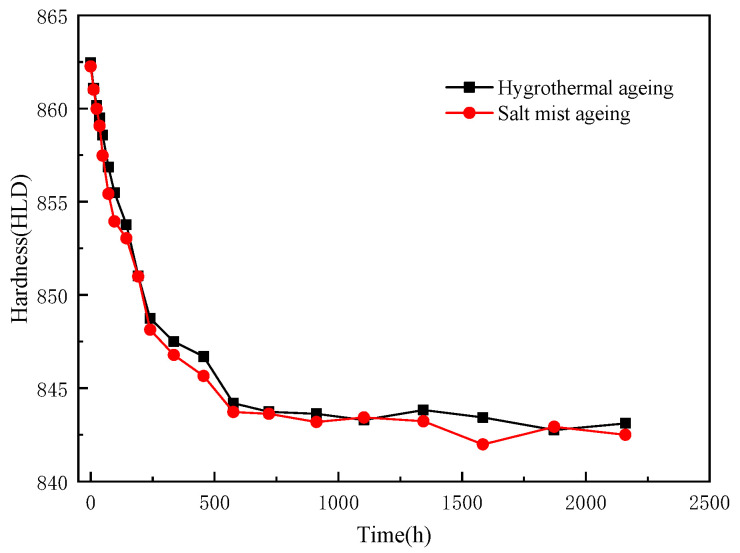
Changes in the hardness of the epoxy resin casting sheets before and after ageing.

The variations in the hardness of the epoxy resins with the ageing time are shown in Figure 8.

The variation in the hardness of the epoxy resins followed a similar trend in the two ageing modes. The hardness gradually decreased over time. The rate of the decrease was initially rapid and then subsequently reduced. The hardness level fluctuated around 843 HLD in the later stages of the ageing. During ageing tests, hardness can indicate the loss of resin properties or the degree of damage. It should be noted that the different ageing methods affected the hardness of the resin at an essentially identical rate. The test depth of the Richter hardness was only approximately 10 μm, and it was mainly determined by the surface property of the resin. Moreover, water penetrates inward from the outside, with water molecules breaching the outer resin and weakening the intermolecular force, thereby decreasing the hardness [26].

#### 3.1.4. Infrared Spectral Analysis

**Figure 9:**
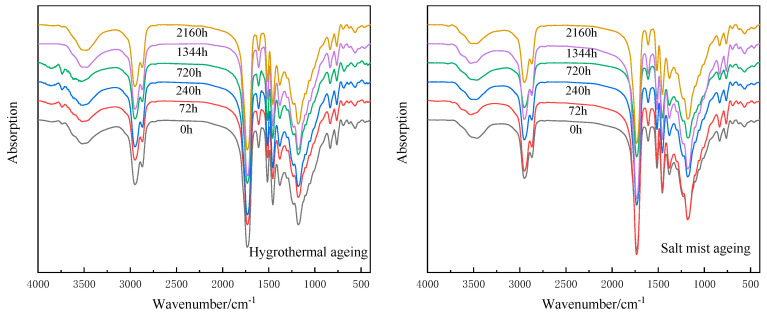
Infrared spectra of the epoxy resin casting sheets after hygrothermal ageing (**left**) and salt mist ageing (**right**).

The infrared spectra of the epoxy resins at different ageing times are shown in Figure 9. The absorption peaks at 2948 cm^−1^ and 2862 cm^−1^ reflect the stretching vibrations of the alkyl units and C−H, respectively; the peak at 1729 cm^−1^ corresponds to the stretching vibration of the C=O; the peaks at 1610 cm^−1^ and 1512 cm^−1^ indicate aromatic structures; the peaks at 1450 cm^−1^ and 1380 cm^−1^ correspond to the bending vibrations of the −CH_3_ and C−H, respectively; the peak at 1180 cm^−1^ corresponds to the stretching vibration of the C−O; and the peak at 829 cm^−1^ is the out-of-plane bending vibration of the Ar−H. The peak positions and intensities of the above chemical light groups did not change significantly before and after the ageing test, indicating that no chemical changes occurred during the ageing process.

The positions and relative intensities of the major absorption peaks in the infrared spectra of the epoxy resin castings before and after ageing were almost identical. This indicates that no significant bond breakage or bond formation occurred during the process and that the two ageing modes did not impact the chemical structure of the resin. The absorption peaks at 1729 cm^−1^ and 1180 cm^−1^, which are attributed to the stretching vibrations of the carbonyl group and ether bond, respectively, indicate the presence of ester groups. The hydrolysis of an ester bond can occur in a humid environment. However, the corresponding changes in the intensities of the absorption peaks shown in Figure 7 at these two positions before and after the ageing did not indicate the occurrence of this reaction. The structure of the benzene ring and cross-linked network increased the steric hindrance of the ester bond and hindered the hydrolysis. Although water permeation can degrade epoxy resin, hydrolysis was not observed in this study. This is because resin degradation is a result of the comprehensive influence of the epoxy chemical composition [56], temperature [24], and pH [22].

### 3.2. Ageing Properties of Epoxy Pultruded Composites

#### 3.2.1. Flexural Strength

**Figure 10:**
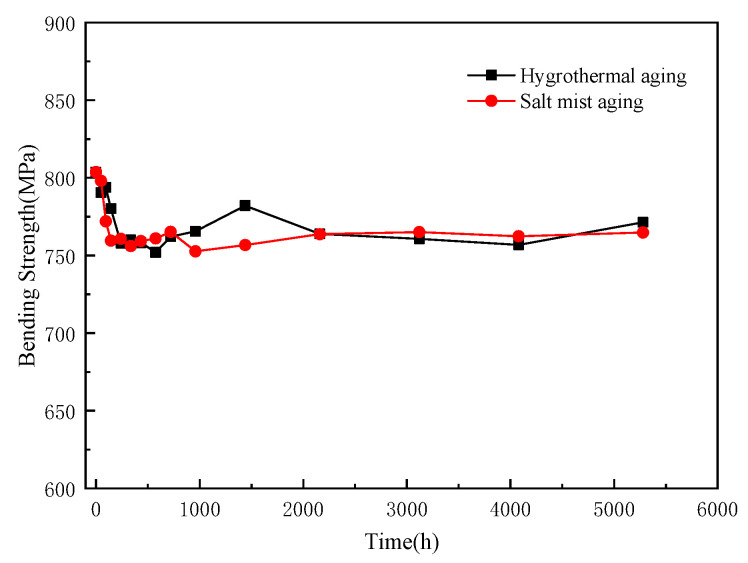
Variations in the flexural strengths of the composites.

The three-point flexural strengths of the epoxy pultruded composites at different ageing times are shown in Figure 10. The flexural strengths of the composites in both the hygrothermal and salt mist ageing conditions follow a similar trend. The initial flexural strength of the composites was 803 MPa. At first, it decreased with an increase in the ageing time, eventually stabilizing at approximately 760 MPa, indicating a decrease of approximately 5.4%.

This implies that the two ageing modes had negligible effects on the bending properties of the composites. The reduction in the flexural strength was primarily due to the swelling of the resin on the material surface, the decreased strength of the intermolecular forces, and the weakening of the matrix–fibre interface caused by water absorption [57]. The hygrothermal and salt mist ageing modes had a minimal effect on the resin matrix, and the water absorbed by the resin did not break the chemical bonds of the resin molecules. Glass and carbon fibre, the reinforcing materials of the composites, act as barriers against water penetration to a certain extent. Therefore, water hardly entered the inner layers of the composites. Hence, the strength of the composites did not decrease continuously. In addition, the outermost layer of the composites was reinforced by glass fibre, which is an insulating material. Consequently, no electrochemical corrosion occurred, and the salt mist ageing mode did not accelerate the decline in the flexural strength.

#### 3.2.2. Impact Strength

**Figure 11:**
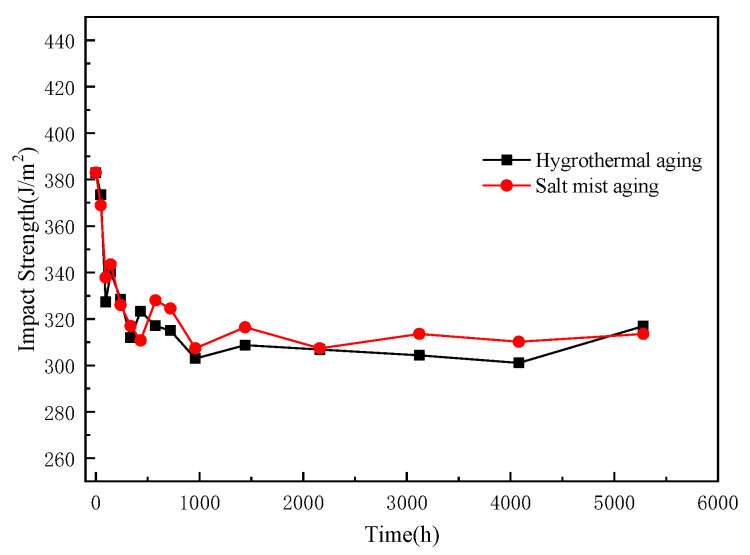
Changes in the impact strengths of the epoxy pultruded composites before and after ageing.

The impact strengths of the epoxy pultruded composites at different ageing times are shown in Figure 11.

The variation in the impact strength was largely similar to that in the flexural strength, although the rate and magnitude of the decrease in the impact strength were relatively large. Alternatively, the impact strength of the composites exhibited a substantially higher sensitivity to ageing. The impact strength of the composite before ageing was 383 J/m^2^, and it stabilized to approximately 310 J/m^2^ in the later stages of ageing, displaying a 19% decrease. The outer layer of the epoxy pultruded composites was a high-strength glass fibre fabric belt, which was classified as a two-dimensional reinforcement that absorbed most of the impact energy during the impact process. The presence of an island-type toughening agent may also increase the toughness of composites in a moist environment [58]. The ageing process primarily affected the outer layer structure of the composites, weakened the bonding between the epoxy resin and the outer fibre, and substantially reduced the impact strength [59].

#### 3.2.3. SEM Analysis

**Figure 12:**
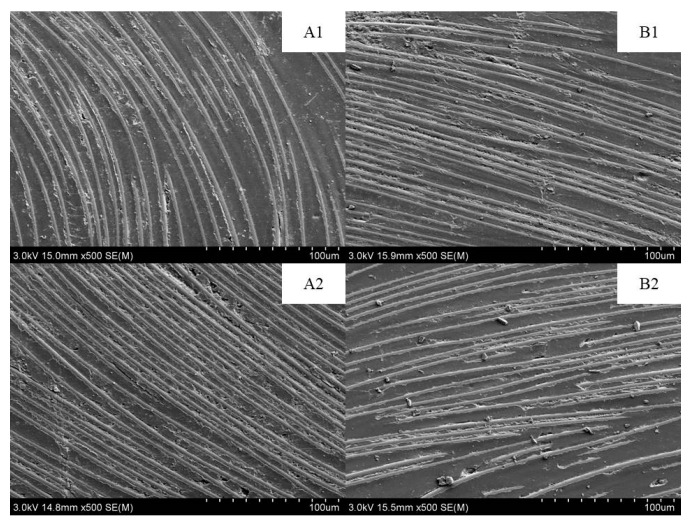
SEM images (at 500× magnification) of the surface morphology of the epoxy pultruded composites before and after ageing ((**A1**,**A2**) hygrothermal ageing, (**A1**) 0 h and (**A2**) 5280 h; (**B1**,**B2**) salt mist ageing, (**B1**) 0 h and (**B2**) 5280 h).

Figure 12 shows the SEM images (at 500× magnification) of the surface morphology of the epoxy pultruded composites at different ageing times. The surface microstructure of the composite materials mostly remained unchanged before and after the hygrothermal and salt mist ageing modes. Moreover, no noticeable damage or flaking of the epoxy resins and fibres was observed. No salt particle deposition was observed on the surface of the materials after the salt mist ageing mode. Furthermore, the salt mist ageing mode showed no corrosive effect on the resin and fibres.

**Figure 13:**
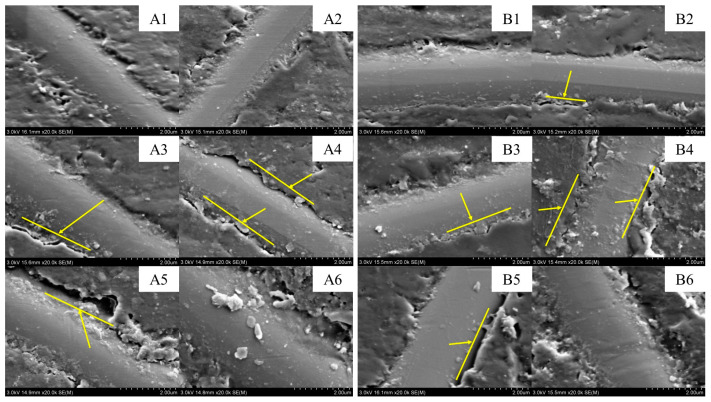
SEM images (at 20k× magnification) of the surface morphology of the epoxy pultruded composites before and after ageing ((**A1**–**A6**) hygrothermal ageing, (**A1**) 0 h, (**A2**) 240 h, (**A3**) 960 h, (**A4**) 2160 h, (**A5**) 3120 h, and (**A6**) 5280 h; (**B1**–**B6**) salt mist ageing, (**B1**) 0 h, (**B2**) 240 h, (**B3**) 960 h, (**B4**) 2160 h, (**B5**) 3120 h, and (**B6**) 5280 h).

Figure 13 shows the SEM images (at 20k× magnification) of the resin–fibre interface in the epoxy pultruded composites at different ageing times. Although the fibre and resin did not change before and after the ageing, micro-cracks appeared at the interface. Before the ageing, the resin and fibre were bonded adequately at the interface; however, the bonding was not very dense. Some micro-pores with a diameter of approximately 0.2 µm existed at the interface. Hence, the interface between the fibre on the outer surface of the composite and the resin matrix was a weak point in the material. The differences in the thermal shrinkage rates of the fibre and resin might result in thermal stress at the interface, substantially shrinking the outer layer and affecting the moisture diffusion [3].

The uneven resin content on the surface of the composites may also have promoted the generation of cracks [60]. The SEM images after 240 h of ageing revealed that micro-cracks began to appear at the interface as the ageing time increased and extended along the axial direction of the fibre. After 3120 h, fractures developed at the edges of the interfacial cracks, and part of the resin broke off. Water entered the composite interface through the micro-pores created by the shrinkage during the curing process. When the amount of water absorbed reached a certain threshold, the resin matrix began to expand and generate stress at the interface. As the water weakened the bonding strength at the interface, cracks formed between the fibre and matrix and then continued to expand along the direction of the interfacial stress.

## 4. Conclusions

To fully grasp the ageing resistance of large-thickness composite products and improve their long-term safety and reliability in the complex outdoor environment, this study was carried out. In this study, the effects of hygrothermal and salt mist ageing on the properties of epoxy/anhydride resins and their carbon fibre/glass fibre-reinforced epoxy pultruded composites were comparatively investigated via artificially accelerated ageing experiments. The results showed that the two ageing modes had similar effects on the properties of the epoxy resins and their epoxy pultruded composites in terms of the manner and degree of impact. The two ageing modes did not cause significant changes to the chemical structure of the epoxy resins, and the ageing process mainly involved physical moisture adsorption. The moisture absorption behaviour of the epoxy obeyed Fick’s law. During the early ageing stage, the moisture absorption rate of the salt mist ageing mode was slightly higher than that of the hygrothermal ageing mode; moreover, during the later stage, the moisture absorption rates gradually declined. After ageing for 2160 h, the moisture saturation rate remained at approximately 1.1% in both modes, and the depth of the moisture penetration across the thickness of the epoxy resin was approximately 0.27 mm. Both ageing modes negligibly affected the thermal decomposition properties and microstructure of the epoxy resins. The hygrothermal and salt mist ageing modes affected the mechanical properties of the epoxy resin composites. The flexural strength and impact strength decreased rapidly during the early ageing stage and then stabilized during the later stage. After ageing for 5216 h, the flexural strength and impact strength of the composites decreased by 5.4% and 19%, respectively, due to the moisture penetrating the interface of the composites and causing micro-cracks between the fibre and resin. The interface between the resin and fibre in the composite material was relatively weak. To further improve the ageing resistance of composites, it is necessary to adopt more effective modification methods to improve the compactness of the composites and enhance the interface bonding strength.

## Figures and Tables

**Table 1:** Salt mist ageing conditions.

One Ageing Cycle (h)	Ageing Temperature (°C)	Concentration of NaCl Solution (g/L)	pH
24	35 ± 2	50 ± 5	6.5~7.2

**Table 2:** Ageing test evaluation time points.

Samples	Evaluation Points/h	Characterisation
Epoxy resin castings	12, 24, 36, 48, 72, 96, 144, 192, 240, 336, 456, 576, 720, 912, 1104, 1344, 1584, 1872, and 2160	Mass change Depth of water penetration Change in colour Density Hardness Infrared spectra Thermogravimetric analysis SEM analysis
Epoxy pultruded composites	48, 96, 144, 240, 336, 432, 576, 720, 960, 1440, 2160, 3120, 4080, and 5280	Flexural strength Impact strength SEM analysis

**Table 3:** Diffusion coefficients under different ageing conditions.

Ageing Test	$M_{\infty }\;(\%)$	*D* (10^−6^ mm^2^/s)
Hygrothermal ageing	1.1	0.98
Salt mist ageing	1.1	1.04

## Data Availability

Not applicable.
